# Pitfalls of Intraoperative Parathyroid Hormone Monitoring in Achieving Complete Surgical Resection of Ectopic Mediastinal Parathyroid Adenoma: A Case Report and Literature Review

**DOI:** 10.7759/cureus.78638

**Published:** 2025-02-06

**Authors:** Wakako Nagase, Yujin Kudo, Jun Matubayashi, Ryosuke Amemiya, Maki Tanigawa, Yoshihisa Shimada, Masaru Hagiwara, Masatoshi Kakihana, Toshitaka Nagao, Tatsuo Ohira, Norihiko Ikeda

**Affiliations:** 1 Surgery, Tokyo Medical University, Tokyo, JPN; 2 Anatomic Pathology, Tokyo Medical University, Tokyo, JPN

**Keywords:** ectopic mediastinal parathyroid adenoma, intact parathyroid hormone, intraoperative parathyroid hormone monitoring, primary mediastinal tumor, video assisted thoracic surgery

## Abstract

Ectopic mediastinal parathyroid adenoma is rare and is generally managed by surgical resection as a definitive treatment. Intraoperative parathyroid hormone (ioPTH) monitoring is valuable for ensuring the complete removal of a target lesion. However, there is no consensus criteria regarding the utilization of ioPTH for complete resection in patients with ectopic mediastinal parathyroid adenomas.

A 65-year-old woman presented with asymptomatic hypercalcemia, and was subsequently diagnosed as having hyperparathyroidism. Radiological imaging displayed a solid mediastinal tumor, suspected to be ectopic mediastinal parathyroid adenoma. Surgical resection was performed together with ioPTH monitoring. Although a transient increase in intact parathyroid hormone (iPTH) level was noted, a 22% decrease in iPTH level compared with the preoperative peak iPTH level was observed 30 minutes after the resection. Intraoperative frozen section diagnosis confirmed complete resection of the ectopic mediastinal parathyroid adenoma. Serum iPTH and calcium levels rapidly decreased postoperatively. The patient was discharged without any complications, and no recurrence was found.

We herein report a case of a patient in whom thoracoscopic removal of an ectopic mediastinal parathyroid adenoma using ioPTH monitoring resulted in a successful postoperative outcome. Our present case demonstrates that although ioPTH monitoring is important, it is also crucial to avoid stimulation of the tumor by intraoperative compression and to confirm complete resection by additional modalities, such as by pathological analysis.

## Introduction

The prevalence of ectopic mediastinal parathyroid adenoma is reported to be 1% to 2% of parathyroid adenomas [1, 2]. Hyperactive tissues within the ectopic lesions secrete an excess amount of parathyroid hormone (PTH), leading to hyperparathyroidism. This condition can result in hypercalcemia or bone mineral disorders, presenting symptoms such as fatigue, bone pain, kidney stones, and gastrointestinal disturbances. Surgical treatment is the first choice for symptomatic primary hyperparathyroidism (pHPT) or asymptomatic pHPT with associated complications [3, 4]. It is particularly noteworthy that in this disease hyperparathyroidism can persist in some patients even after treatment. Furthermore, surgical management techniques that are required for these patients might be unfamiliar to thoracic surgeons who have limited experience with this disease.

Inadequate resection of the hyperfunctioning parathyroid gland is a major issue in surgical treatment for ectopic mediastinal parathyroid adenoma, which can result in inefficient postoperative improvement of pHPT. It may be difficult to identify active parathyroid glands intraoperatively, particularly when they are located deep within the mediastinum or thymus. Intraoperative parathyroid hormone (ioPTH) monitoring has been reported to be a valuable technique for confirming complete tumor resection [5-11]. Although the technique is widely used, and various criteria for the use of ioPTH monitoring have been proposed, a universally accepted method has not yet been established. Therefore, understanding the pitfalls of this technique is crucial to making an accurate decision of complete resection of the responsible tumor. We here report a case of successful thoracoscopic removal of an ectopic mediastinal parathyroid adenoma using ioPTH monitoring, together with a review of previously reported cases.

## Case presentation

A 65-year-old woman presented with asymptomatic hypercalcemia that was identified during a routine medical check-up. She had a past medical history of kidney stones, cerebrovascular disease, hypertension, arrhythmia, and hyperlipidemia. Laboratory examination results included a serum calcium level of 12.7 mg/dL, serum phosphate concentration of 1.9 mg/dL, serum alkaline phosphatase activity of 156 IU/L, serum albumin level of 4.2 g/dL, and serum intact parathyroid hormone (iPTH) level of 331 pg/mL. Her renal function was within the normal range, with a serum creatinine level of 0.86 mg/dL and an estimated glomerular filtration rate (eGFR) of 51.0 (mL/min/1.73 m^2^) (Table 1). Her bone density measured by dual-energy X-ray absorptiometry scan demonstrated a young adult mean of 73%. From these results, she was diagnosed as having primary hyperparathyroidism.

**Table 1:** Laboratory data on admission AST: aspartate aminotransferase, ALT: alanine transaminase, LDH: lactate dehydrogenase,  γ-GTP: gamma-glutamyl transpeptidase, ALP: alkaline phosphatase, T-bil: total bilirubin, Alb: albumin, Glu: glucose, BUN: blood urea nitrogen, Cre: creatinine, eGFR: estimated glomerular filtration rate, Na: sodium, Cl: chloride, K: potassium, Ca: calcium, P: phosphate, CRP: C-reactive protein, PTH: parathyroid hormone, TSH: thyroid stimulating hormone, FT3: free triiodothyronine, FT4: free thyroxine

Complete blood count		Normal range	
White blood cells	6,900	2,700-8,800	/μl
Red blood cells	370x10^4^	370-540x10^4^	/μl
Platelet	21x10^4^	14-34.0x10^4^	/μl
Blood chemistry			
AST	16	8-38	U/l
ALT	15	4-44	U/l
LDH	146	106-211	U/l
γ-GTP	30	16-73	U/l
ALP	156	104-338	U/l
T-Bil	0.37	0.20-1.20	mg/dl
Alb	4.2	3.9-4.9	g/dl
Glu	90	60-110	mg/dl
BUN	19.9	8.0-22.6	mg/dl
Cre	0.86	0.40-0.80	mg/dl
eGFR	51.0		ml/min/1.73m^2^
Na	141	138-148	mmol/l
Cl	110	98-108	mmol/l
K	4.5	3.6-5.2	mmol/l
Ca	12.7	8.2-10.2	mg/dl
P	1.9	2.5-4.7	mg/dl
CRP	<0.02	<0.30	mg/dl
Endocrine examination			
Intact-PTH	331	10-65	pg/ml
TSH	1.81	0.50-5.00	μIU/ml
FT3	2.38	2.30-4.30	pg/ml
FT4	1.23	0.90-1.70	ng/dl

**Figure 1:**
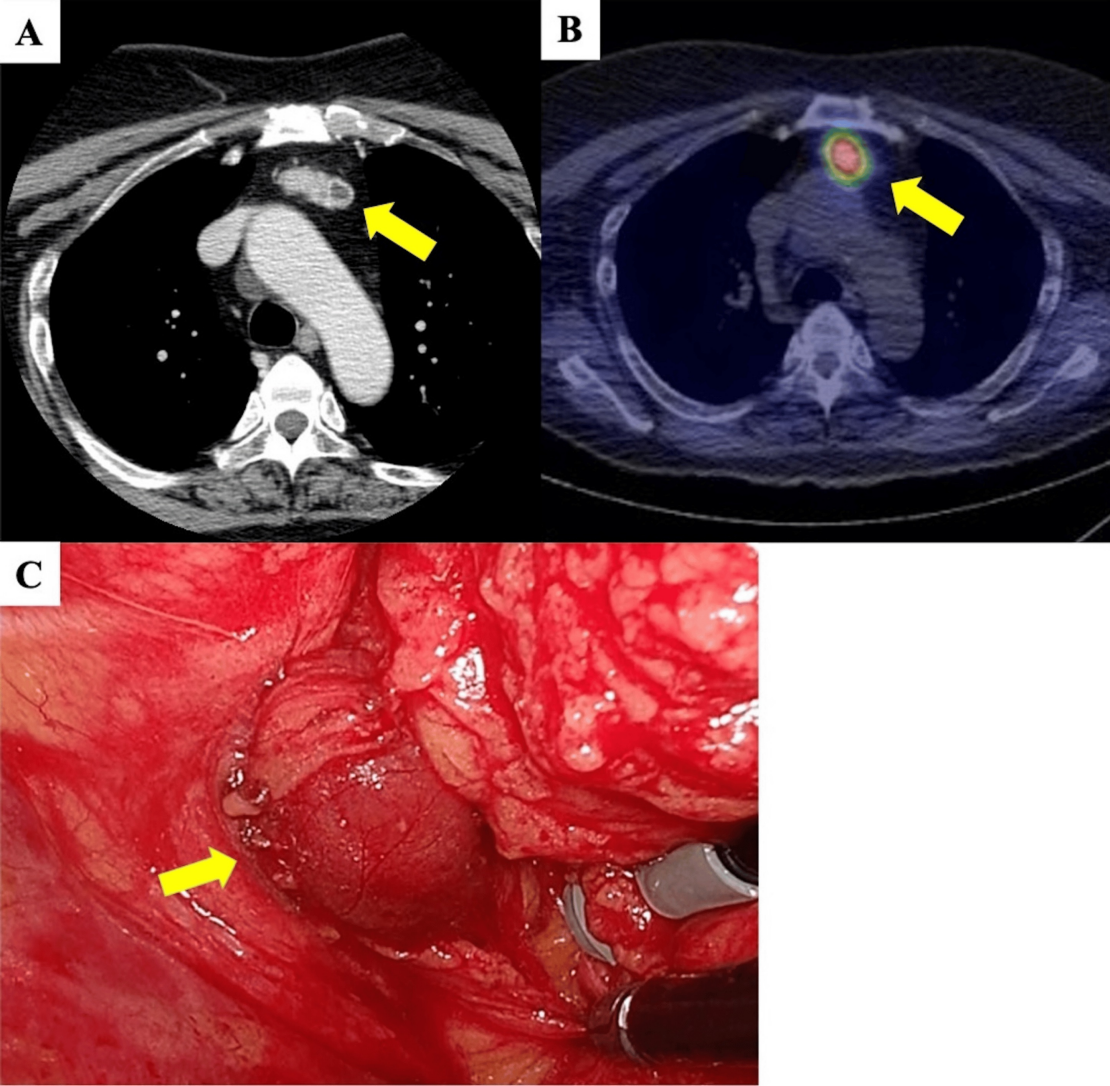
Chest computed tomography (CT) and technetium-99m-methoxy-isobutyl-isonitrile (99mTc-MIBI) scintigraphy and intraoperative findings of the patient (A) Chest CT displayed a 3.3 x 1.4-cm solid mass containing a cyst in the anterior mediastinum (yellow arrow); (B) 99mTc-MIBI scintigraphy displayed increased uptake within the mass (yellow arrow); (C) A round tumor was observed in the anterior mediastinum (yellow arrow).

Chest computed tomography displayed a 3.3 x 1.4 cm solid mass containing a cyst in the anterior mediastinum (Figure 1A). Technetium-99m-methoxy-isobutyl-isonitrile scintigraphy displayed increased uptake within the mass (Figure 1B). Ultrasound examination of the thyroid did not display any visibly enlarged parathyroid glands. Based on these findings, the lesion was suspected to be an ectopic mediastinal parathyroid tumor, which was causing the pHPT. Surgical resection of the anterior mediastinal tumor was performed using the 4-port video-assisted thoracoscopic surgery (VATS) approach. Intraoperatively, it was initially difficult to identify the tumor owing to the abundant mediastinal fat tissue and the limited space in the anterior thoracic cavity. The phrenic nerve was preserved, and the tumor was identified in the thymic tissue (Figure 1C). 

**Figure 2:**
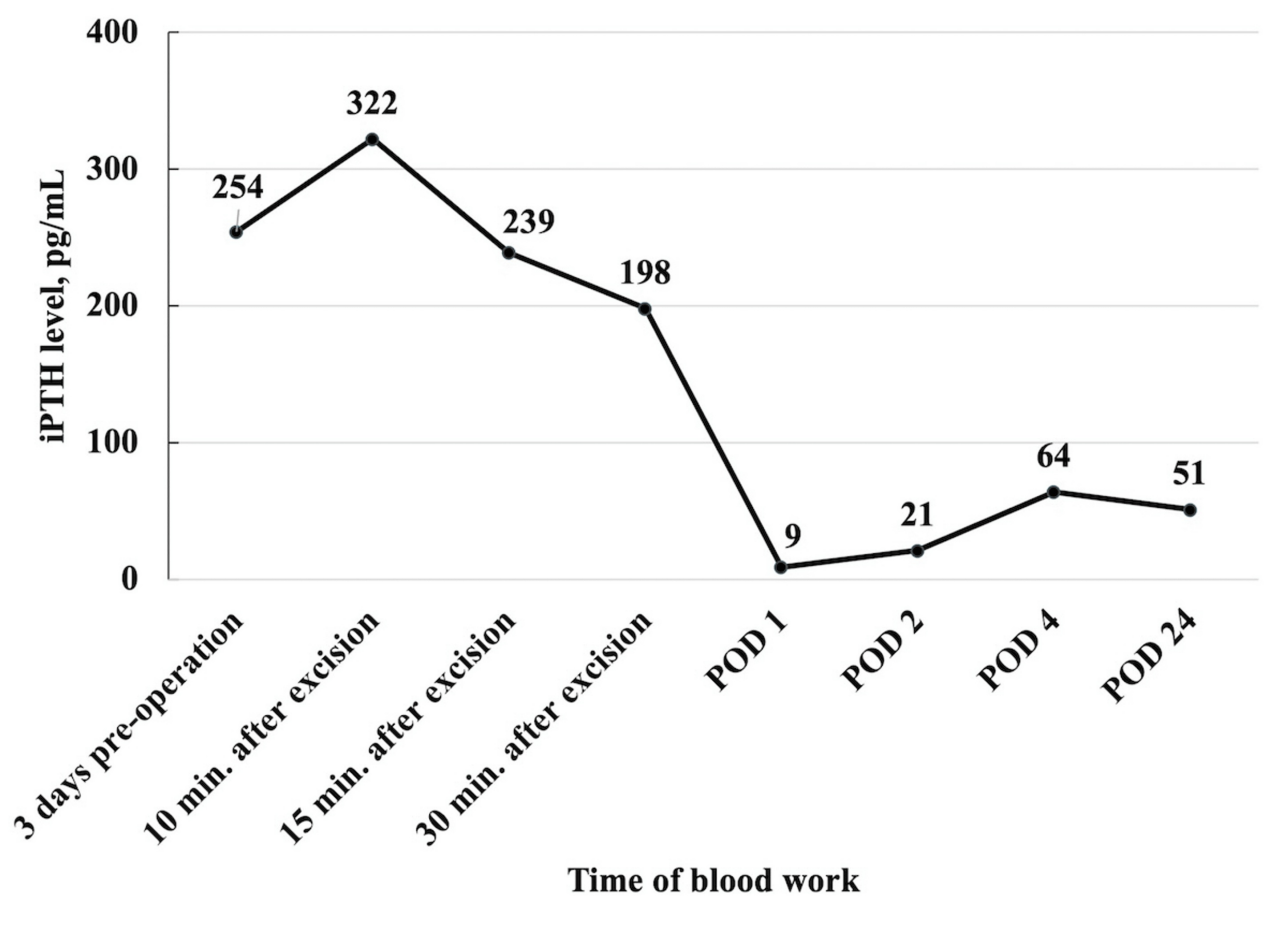
Results of iPTH measurements A 22% decrease in iPTH level compared with the preoperative iPTH level was observed at 30 minutes after excision of the parathyroid gland. The iPTH level immediately decreased to within the normal range on postoperative day 1.

The tumor was resected en bloc along with the surrounding mediastinal fatty tissue. There is a possibility that the tumor, together with the mediastinal fatty tissue, was compressed when trying to obtain a clear view of the operative field to safely identify the anatomical landmarks.. The levels of ioPTH were measured by drawing peripheral blood at four-time points, namely, five minutes, 10 minutes, 15 minutes, and 30 minutes after removing the ectopic parathyroid tumor. There was a transient increase in the level of ioPTH from 254 to 322 pg/mL five minutes after the resection of the ectopic parathyroid tumor. However, the ioPTH level decreased thereafter, and a 22% reduction compared with the preoperative iPTH level was observed 30 minutes after tumor resection. Details of the iPTH levels are shown in Figure 2. Intraoperative frozen section diagnosis confirmed that the lesion was an ectopic mediastinal parathyroid adenoma.

**Figure 3:**
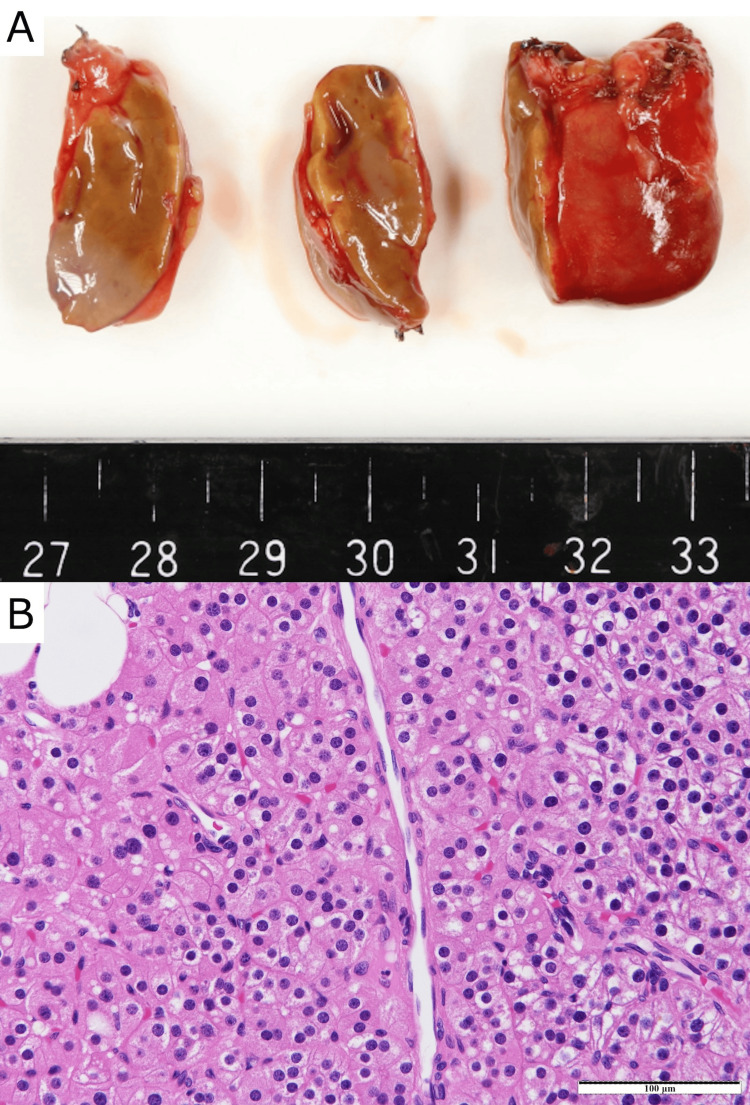
Macroscopic and microscopic appearance of the tumor (A) Glossy, the excised tumor measured 3.3×2.3×1 cm in size and appeared to be a well-circumscribed mass with a thin fibrous capsule; (B) Microscopically, the tumor was composed of cells arranged in cord- and nest-like fashion. Many tumor cells with small round nuclei and clear or eosinophilic cytoplasms were observed. (Hematoxylin–Eosin staining, × 200 magnification).

Glossy, the excised tumor measured 3.3×2.3×1 cm in size and appeared to be a well-circumscribed mass with a thin fibrous capsule (Figure 3A). Microscopically, the tumor was composed of cells arranged in cord- and nest-like fashion. Many tumor cells with small round nuclei and clear or eosinophilic cytoplasms were observed (Figure 3B). Therefore, the lesion was pathologically diagnosed as parathyroid adenoma. On the day after the operation, the patient’s iPTH level immediately decreased to within the normal range. The patient’s serum calcium level improved to 9.8 mg/dL on the second day after the operation. No signs of hypocalcemia were detected upon careful monitoring. The patient was discharged without any postoperative complications. There was no occurrence of hypocalcemia during the postoperative follow-up period of 1.5 years.

## Discussion

In the present case, the patient had an ectopic mediastinal parathyroid tumor and presented with primary hyperparathyroidism, and the tumor was resected by VATS together with ioPTH monitoring. Unexpectedly, intraoperative manipulation and compression of the tumor resulted in an initial temporary increase in iPTH level, and the expected significant decrease in iPTH level after resection was not observed. The surgery was completed after intraoperative frozen section analysis confirmed that tumor resection was complete, and the iPTH level decreased on postoperative day one. Our present case demonstrates that upon resection of an ectopic mediastinal parathyroid tumor in a patient with primary hyperparathyroidism, it is crucial to minimize manipulation of the tumor, as compression of the tumor can cause an increase in iPTH level, leading to unreliable intraoperative monitoring results.

The use of ioPTH monitoring has been demonstrated to be beneficial for the resection of ectopic mediastinal parathyroid adenomas [9-12]. Sagan et al. demonstrated the utility of ioPTH monitoring, demonstrating a decrease in the potential failure rate from 21.2% without ioPTH monitoring to an actual 3% when ioPTH was utilized during the surgery [9]. Monitoring of ioPTH provides surgeons with a real-time assessment of parathyroid function and helps to ensure complete removal of the responsible lesion. Although ioPTH monitoring is routinely used and there are several criteria, there are no established standard criteria that have achieved universal consensus. Prior studies have demonstrated a decrease in ioPTH level of more than 50% from the baseline as a valuable criterion for complete resection of primary parathyroid tumors [13, 14]. With regard to ioPTH monitoring, particularly for mediastinal parathyroid tumors, there is no consensus on the degree and timing of PTH reduction that indicates successful surgery.

We performed a literature review of patients who underwent ectopic mediastinal parathyroid tumor resection together with ioPTH monitoring. A literature search was conducted with the keywords [(parathyroid adenoma) OR (parathyroid tumor)] AND (mediastinum OR mediastinal OR (ectopic mediastinal)] by a medical subject heading (MeSH) search in PubMed®. The time frame was set from 1996 to 2023 because rapid PTH measurement became commercially available in 1996. Of the 384 case reports identified, we selected appropriate cases as follows: (1) surgically resected mediastinal parathyroid tumors with ioPTH monitoring reported in detail, (2) reports with less than two cases to avoid overlapping reports, (3) articles in English.

In our comprehensive literature review, a total of 18 cases were identified and analyzed (details are shown in Table 2). The extent of ioPTH reduction and the timing of ioPTH sampling are particularly noteworthy. We found that 17 patients showed a reduction in ioPTH levels by more than 50% from the initial levels. The decrease was approximately 80% to 90% in most cases. There was one patient (case five) in whom the ioPTH reduction rate was less than 50% after the initial resection of pericardial fat tissue in the anterior mediastinum. Consequently, further neck exploration was performed, resulting in the excision of an enlarged parathyroid gland and a 67% reduction in ioPTH levels five minutes post-excision. In such cases, the use of the “Wisconsin Criteria” is recommended, which requires that the PTH value is at least 50% lower than the pre-excision level at either five, 10, or 15 minutes post-excision if the PTH at five minutes is increased from the baseline, that number becomes the “new baseline”, and a sample is drawn 20 minutes from this timepoint [15]. However, in our present case, a transient increase in ioPTH levels was observed, without a marked subsequent decrease during the operation. This could be caused by inadvertent compression of the tumor owing to the presence of abundant mediastinal fat during the surgery.

**Table 2:** Previously reported cases of patients who underwent surgical resection of ectopic mediastinal parathyroid adenoma together with ioPTH monitoring a) The level of decrease from the baseline iPTH level that was first seen as a more than 50% decrease. Baseline iPTH level was defined as the level pre-excision, including blood work performed at pre-operation, b) Blood test performed at the first tumor excision.

No.	Study	Year	No. of patients	Age (years)	Sex	Tumor location in mediastinum	Surgical approach	Diagnosis	iPTH Reduction rate^a^	Time of blood work after tumor excision
1	O’Herrin et al. [16]	2002	1	43	F	Anterior	VATS	Hypercellular parathyroid	87%	5 min
2	Luncă at al. [17]	2004	1	44	F	Middle	Sternotomy	Parathyroid adenoma	70%	20 min
3	Profanter at al. [18]	2004	1	57	F	Superior	RATS	Parathyroid adenoma	84%	10 min
4	Amar et al. [19]	2004	2	36	F	Anterior	VATS	Parathyroid tumor	97%	Not indicated
				57	F	Anterior	VATS	Adenoma or hyperplasia	78%	Not indicated
5	Wild et al. [20]	2006	1	65	M	Anterior	VATS	Adenoma	30%	5 min^b^
6	Moreno et al. [21]	2007	1	69	F	Middle	VATS	Parathyroid gland	95%	Post operation
7	Kim et al. [22]	2014	1	59	F	Anterior	VATS	Parathyroid adenoma	94%	Not indicated
8	Schwarzlmüller et al. [23]	2014	2	72	F	Middle	Sternotomy	Adenoma	96%	10 min
				79	F	Middle	Sternotomy	Adenoma	97%	10 min
9	Dinga Madou et al. [24]	2016	1	55	F	Middle	Sternotomy	Adenoma	68%	0 min
10	Elhelf et al. [25]	2017	1	58	M	Superior	Mediastinoscopy	Hypercellular parathyroid tissue with minute thymic remnant	57%	10 min
11	Ishikawa et al. [26]	2017	1	66	F	Superior	VATS	Adenoma	85%	10 min
12	Mitsuboshi et al. [27]	2019	1	67	F	Anterior	Thoracotomy	Adenoma	87%	15 min
13	Medbery et al. [28]	2019	1	61	M	Posterior	Thoracotomy	Adenoma	73%	5 min
14	Miller et al. [29]	2019	1	53	M	Posterior	Transcervical	Adenoma	53%	5 min
15	Iijima et al. [12]	2022	1	53	F	Anterior	RATS	Adenoma	52%	15 min
16	Akin et al. [30]	2022	1	48	F	Superior	Transcervical	Adenoma	86%	10 min

**Figure 4:**
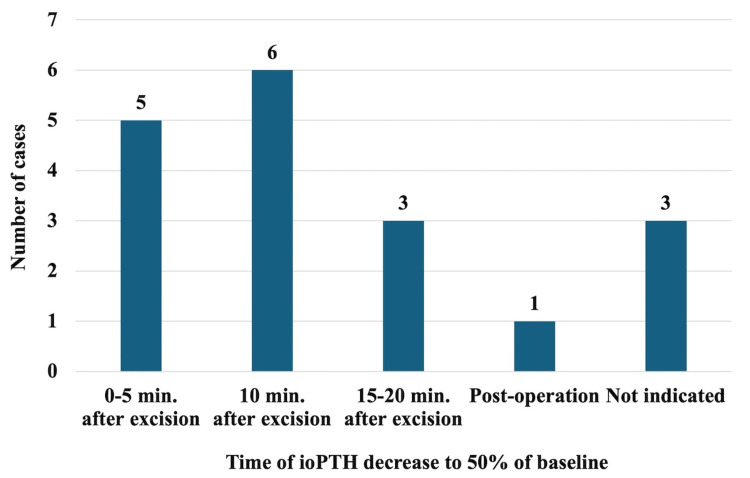
Timepoints at which ioPTH values decreased to below 50% of baseline (pre-operation or pre-excision) in cases from the literature review The most common time to observe a decrease in ioPTH levels to below 50% of baseline was 5 to 10 minutes after tumor excision.

Another crucial factor is the timing of the blood tests. As shown in Figure 4, the most common time at which ioPTH levels were observed to decrease to below 50% of the baseline was five to 10 minutes after tumor resection. This was owing to the inherent characteristic of PTH having a short half-life (about three to five minutes) [3]. Previous reports have indicated potential pitfalls in ioPTH monitoring. An increase in the ioPTH level five minutes after tumor resection, or an ioPTH spike may result in false-negative results of ioPTH monitoring [15-17]. In our patient, ioPTH levels were measured up to 30 minutes after tumor resection, but no decrease below 50% of the baseline was observed. However, due to the findings of preoperative imaging suggesting a single responsible active gland and the diagnosis of a pathological frozen section diagnosis confirming complete resection of the tumor, we considered that the surgery was accomplished. Studies have mentioned that when pressure is applied to a parathyroid gland, it triggers the release of hormones, leading to an elevation in peripheral blood levels [5]. The transient increase in ioPTH levels above the preoperative baseline in this patient was likely caused by compression of the tumor during surgery, which subsequently decreased over time.

## Conclusions

We successfully resected an ectopic mediastinal parathyroid adenoma from a patient, but a sufficient reduction in ioPTH level could not be confirmed intraoperatively. Although ioPTH monitoring is an important indicator of the complete resection of ectopic mediastinal parathyroid adenomas, it is very important to be aware that the surgical technique has the potential to inadvertently increase ioPTH levels, which may make the evaluation difficult. Serum PTH has a short half-life of three to five minutes, necessitating a comprehensive evaluation of the result. Careful ioPTH monitoring procedures and appropriate surgical techniques are the key to the successful treatment of ectopic mediastinal parathyroid adenomas.
